# Modulating Persistent Photoconductivity through Barrier Engineering for High‐performance and Multifunctional Two‐dimensional Optoelectronic Devices

**DOI:** 10.1002/advs.202524279

**Published:** 2026-03-03

**Authors:** Panpan Huo, Xinhao Zhang, Xiangyong Cui, Yeming Wang, Dong Zhou, Baoyuan Man, Il Jeon, Won Jong Yoo, Cheng Yang

**Affiliations:** ^1^ School of Physics and Optoelectronics Shandong Normal University Jinan People's Republic of China; ^2^ Department of Nano Engineering Department of Nano Science and Technology SKKU Advanced Institute of Nanotechnology (SAINT) Sungkyunkwan University (SKKU) Suwon Republic of Korea; ^3^ SKKU Global Research Center (SGRC) Sungkyunkwan University (SKKU) Suwon Republic of Korea; ^4^ Shandong Provincial Key Laboratory of Light Field Manipulation Physics and Applications Jinan People's Republic of China

**Keywords:** 2D materials, barrier engineering, persistent photoconductivity, photodetectors, photomemory devices

## Abstract

2D optoelectronic devices, such as photomemory and photodetectors, have exceptional potential for realising multifunctional applications. Modulating persistent photoconductivity (PPC) is key to enabling diverse functionalities and optimising device performance. However, PPC in 2D devices is difficult to modulate due to surface defects (trap states), Fermi level pinning, and the Schottky barrier height at junctions. In this study, we demonstrate that reducing surface defects and Fermi level pinning enables barrier engineering for effective PPC modulation. The persistent photoconductivity gain (PPCG) is tunable from 307.6% to 4.72%, enabled by tailoring the Schottky barrier height through a van der Waals contact strategy. A high PPCG (307.6%) is achieved in a high‐barrier Au/MoS_2_ junction, suitable for optoelectronic synapses, neuromorphic computing, and photomemory applications, with a relaxation current linearity of up to 0.986. A low PPCG (4.72%) is achieved in a low‐barrier 1T′‐WTe_2_/MoS_2_ junction, designed for photodetectors with an on/off ratio of 10^5^, a response time of 2 ms, and a responsivity of 30.1 A/W. These findings advance the understanding of PPC and provide new avenues for designing high‐performance 2D optoelectronic devices.

## Graphical abstract

Schottky barrier engineering using van der Waals contacts enables effective modulation of persistent photoconductivity (PPC) in optoelectronic devices. A high persistent photoconductivity gain (PPCG) (307.6%) is achieved in a high‐barrier Au/MoS_2_ junction, supporting photomemory and optoelectronic synaptic behaviors. A low PPCG (4.72%) is achieved in a low‐barrier 1T′‐WTe_2_/MoS_2_ junction, enabling fast, stable photodetectors with minimal signal drift.

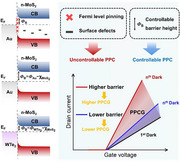

## 1 Introduction

2D optoelectronic devices are strong candidates for post‐silicon technologies in photomemory, high‐performance photodetectors, and flexible electronics, owing to their tunable optoelectronic properties, high carrier mobility, and fast response times [1, 2, 3, 4, 5]. While most studies have focused on specific device types, the interrelationship between different devices has been underexplored. Persistent photoconductivity (PPC) is a critical parameter in designing optoelectronic devices for various applications. PPC is a light‐induced phenomenon in which photoconductivity persists even after light excitation is terminated [6]. High and stable PPC significantly enhances data storage efficiency and processing capability in photomemory and neuromorphic computing applications. In contrast, low PPC improves response speed and signal accuracy in photodetectors [7, 8, 9, 10]. Consequently, controllable PPC opens novel avenues for designing high‐performance, multifunctional optoelectronic devices.

Various methods have been studied to modulate PPC. Hydrophobic surfaces have been employed to suppress random localized potential fluctuations [11]. Short‐duration voltage pulses have been applied to induce electron accumulation and accelerate recombination [12]. Thermal relaxation processes have been introduced to reduce dark current and shorten response times [13]. Water treatment has been used to remove hydrogen from the material surface, thereby reducing internal electric fields and promoting electron relaxation [14, 15].

However, significant challenges remain in effectively modulating PPC: (1) most existing methods depend on external conditions and environmental factors, which are difficult to control; (2) intrinsic material properties and interface engineering have been largely overlooked, limiting precise and flexible PPC regulation; and (3) there is a lack of effective strategies to ensure long‐term stability and reproducibility of PPC modulation.

In this paper, we present a novel barrier engineering strategy for modulating PPC. Au/MoS_2_ and 1T′‐WTe_2_/MoS_2_ junctions were fabricated by dry transfer of exfoliated flakes onto thoroughly cleaned SiO_2_/Si substrates with pre‐patterned Au electrodes. The weakly interacting bottom contact forms a van der Waals (vdW) interface, minimising surface defects and mitigating Fermi level pinning, so that the Schottky barrier height becomes the primary variable controlling PPC. Schottky barrier heights of 754 and 473 meV are achieved in Au/MoS_2_ and 1T′‐WTe_2_/MoS_2_ junctions, respectively, enabling modulation of the persistent photoconductivity gain (PPCG) from 307.6% to 4.72%. High‐performance photomemory (relaxation current linearity of 0.986) and photodetectors (on/off ratio of 10^5^) are successfully demonstrated.

## 2 Results

### 2.1 Strategy for Modulating PPC via Controllable Schottky Barrier Height

**FIGURE 1:**
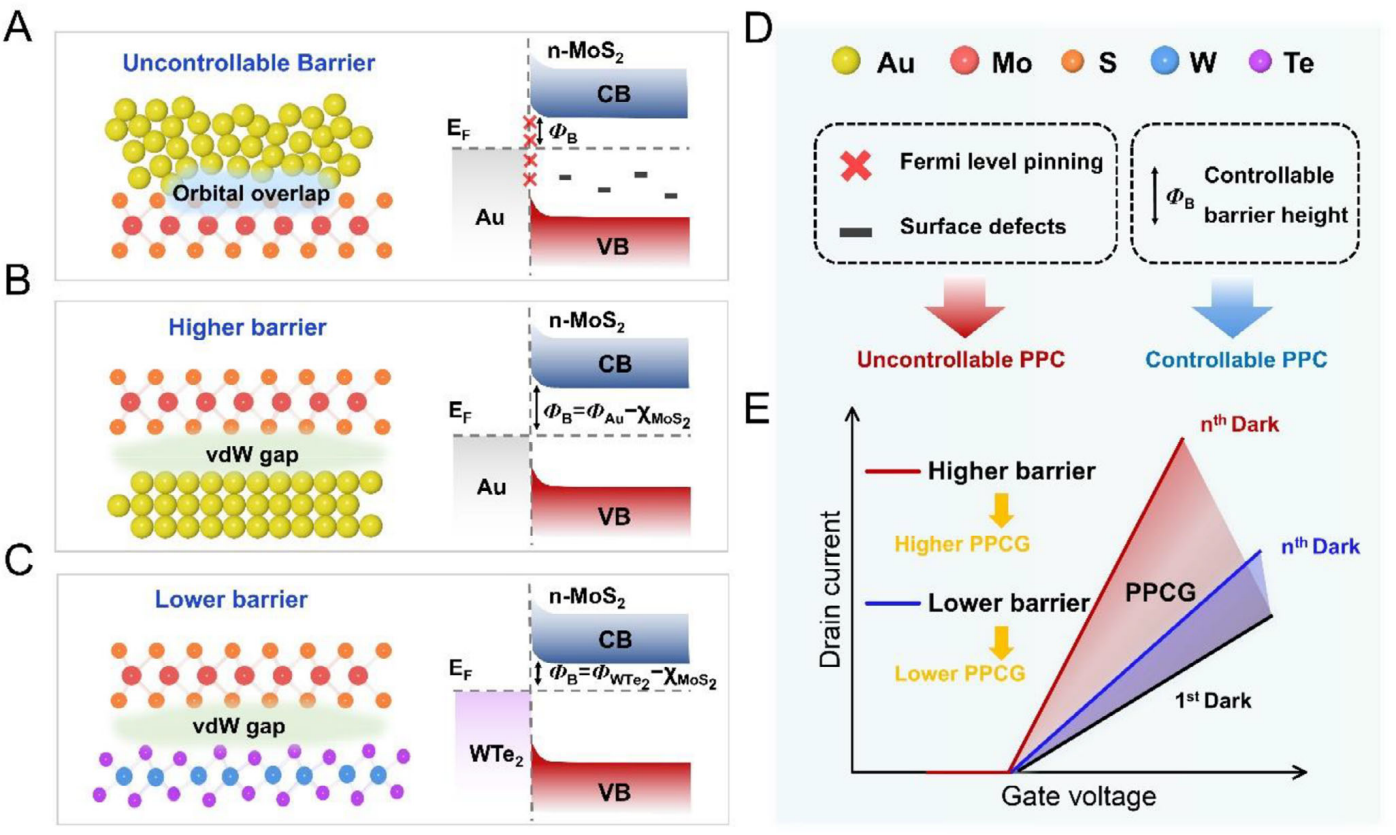
Strategy for modulating PPC via controllable Schottky barrier height. Schematics of (A) uncontrollable Schottky barrier height, (B) controllable high Schottky barrier height, and (C) low Schottky barrier height. (D) Schematic showing factors influencing the PPC phenomenon. (E) Schematic of PPC modulation via controllable Schottky barrier height. The black line represents the current under initial dark conditions; the red and blue lines indicate the current after the n^th^ light–dark cycle. PPCG denotes the PPC gain, used to quantify the magnitude of PPC.

In general, the Schottky barrier height at 2D semiconductor/3D metal junctions is uncontrollable. For industrial‐scale production, 3D metals are typically deposited via electron beam evaporation or sputtering using photolithography. This process irreversibly damages the atomic structure of 2D materials, introducing surface defects [16], dangling bonds, impurities, or oxide layers at the interface, as illustrated in Figure 1A. Orbital overlap at the interface readily induces Fermi level pinning, in which the Fermi level becomes fixed at a certain energy, largely independent of the work function of the metal. Consequently, the Schottky barrier height deviates from the Schottky–Mott relationship. This non‐ideal behavior, coupled with the random density and distribution of surface defects, renders the Schottky barrier height intractable.

Several methods have been explored to achieve a controllable Schottky barrier height. Interface engineering, such as inserting buffer layers or passivation, reduces interface states and mitigates pinning effects [17]. Doping techniques, including n‐type doping near the interface, modify carrier concentrations to adjust the barrier height [18]. Surface treatments such as plasma exposure or self‐assembled monolayers can alter the work function to tune the Schottky barrier [19]. More recently, vdW contacts have been investigated to form clean metal–semiconductor interfaces [20, 21]. These interfaces better adhere to the Schottky–Mott relationship, enabling controllable high and low Schottky barriers, as shown in Figure 1B, C.

Under illumination, electron–hole pairs are generated at the interface. However, surface defects may trap carriers, forming localised states that impede the efficient separation and transfer of photogenerated carriers. Additionally, Fermi level pinning affects charge transport and recombination dynamics. After light exposure, electrical conductivity remains elevated for an extended period, which is the PPC phenomenon. When surface defects and Fermi level pinning are uncontrolled, PPC also becomes difficult to control, as shown in Figure 1D.

These defects and pinning effects cause several issues [22, 23]. Excessive carrier trapping slows recombination, reducing photodetector response speed. Moreover, PPC varies across nominally identical devices due to fabrication‐induced variations in defect formation. Even a single device may exhibit inconsistent PPC behavior across repeated measurements, reflecting the uncontrolled evolution of surface defects.

By addressing surface defects and Fermi level pinning, a controllable Schottky barrier height can be achieved, enabling precise barrier engineering and effective PPC modulation. Schottky barrier height directly governs the transport and recombination dynamics of photogenerated carriers, thus controlling the PPC behavior.

We introduce a new quantitative metric, persistent photoconductivity gain (PPCG), to characterise the magnitude of PPC, as shown in Figure 1E. We propose that a higher Schottky barrier height leads to a higher PPCG, while a lower Schottky barrier height results in a lower PPCG. To verify the reproducibility of barrier engineering between devices, we characterised multiple independently fabricated devices for each structure. Their transfer characteristics and extracted Schottky barrier heights showed consistent distributions (Figure S1), confirming that the high‐barrier (Au/MoS_2_) and low‐barrier (1T′‐WTe_2_/MoS_2_) junctions are reproducible rather than coincidental.

### 2.2 Strategy for Addressing the Surface Defects and Fermi Level Pinning Effects

**FIGURE 2:**
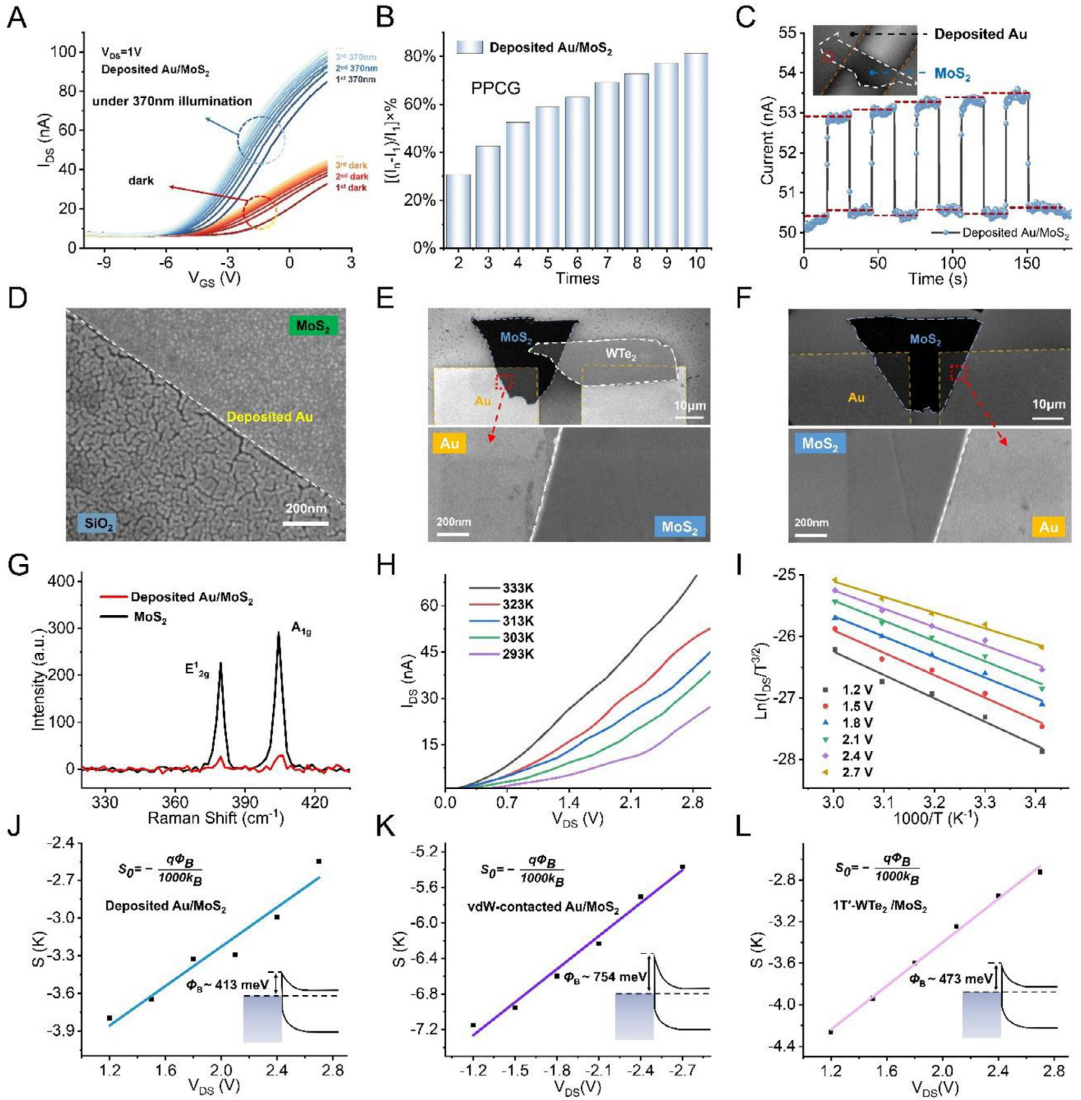
Strategy for addressing the surface defects and Fermi level pinning effects. (A) Dark–light test of the deposited Au/MoS_2_ devices. (B) PPCG for deposited Au/MoS_2_ devices. (C) Time response of deposited Au/MoS_2_ devices, the red dashed box is the magnified area in Figure 2D. SEM images of MoS_2_ surface morphology at the same magnification: (D) Au‐deposited MoS_2_, (E) vdW‐transferred MoS_2_ on WTe_2_/Au/SiO_2_, (F) vdW‐transferred MoS_2_ on Au/SiO_2_. (G) Raman spectra of MoS_2_ films before and after Au deposition. (H) *I*_ds–_ *V*_ds_ curves of deposited Au/MoS_2_/Au FET at various temperatures. (I) Arrhenius plots of the deposited Au/MoS_2_/Au FET at various *V*_ds_. Relationships between slopes from Arrhenius fitting and *V*_ds_ for (J) Au/MoS_2_/Au FET, (K) vdW‐contacted Au/MoS_2_/Au FET, and (L) vdW‐contacted Au/MoS_2_/WTe_2_/Au FET.

The PPC in the deposited Au/MoS_2_ device, being governed by defect‐related traps, is poorly controllable. To determine the potential PPC effect in the deposited Au/MoS_2_ structure, we measured the transfer electrical characteristics (*I*_ds–_ *V*_gs_) curves of the device under dark and 370 nm light conditions. By conducting several cycles of this dark–light test, we aimed to understand the stability of the device under illumination. As shown in Figure 2A, the deposited Au/MoS_2_ device exhibits pronounced defect‐dominated behavior. Under illumination, photogenerated carriers are partially trapped at interfacial states, and their slow release after the light is turned off prevents the channel from fully returning to its initial state. Therefore, the subsequent transfer scans are performed on a channel that is still effectively photodoped by the residual trapped charges, leading to an upward shift of both the photocurrent and dark current curves with increasing cycle number. At the same *V*_gs_ and *V*_ds_, the current progressively increases, corresponding to reduced resistance and enhanced conductivity, which is characteristic of PPC [12, 24, 25]. As the number of cycles further increases, the trap states gradually become filled, and the increase in current correspondingly decreases and eventually stabilizes.

To quantitatively assess PPC in the deposited Au/MoS_2_ structure, the previously defined PPCG metric is used. PPCG is defined as [(*I*_n_‐*I*_1_)/*I*_1_] × 100%, where *I*_1_ is the initial dark current and *I*_n_ is the dark current after the n^th^ measurement cycle. All currents used for PPCG evaluation were extracted at a fixed drain–source voltage of *V*_ds_ = 1.0 V. For the gate voltage *V*_gs_, PPCG was calculated at three discrete voltage points selected within the rising region of the transfer curve, and the final PPCG value was obtained by averaging the results from these points to minimise uncertainties associated with a single voltage point. Unlike qualitative descriptions, PPCG provides a clear, systematic measure of PPC across different device configurations. As shown in Figure 2B, the deposited Au/MoS_2_ exhibits a PPCG of up to ∼80% after stabilization. However, since the degree of defects in the device fabrication process is not deterministically controlled, the PPCG due to defects will also vary, inevitably leading to uncontrollable PPC. In addition, due to defect‐dominated PPC causing intrinsic uncontrollability, even when the PPC response is near saturation, the dark current remains highly unstable, which will affect the measurement of photocurrent and thus hinder the further application of MoS_2_‐based devices in optoelectronics (Figure 2C).

Surface defects are readily introduced on the surface of 2D materials when traditional sputtering deposition techniques are used to deposit 3D metals. A significantly higher unit grain density is observed on MoS_2_‐supported Au films compared with Au films on SiO_2_, as shown in Figure 2D. During Au deposition on few‐layer MoS_2_ flakes, energetic Au atoms generate defects in the atomically thin 2D material. These defects enhance atomic adsorption and promote a high nucleation density [26, 27]. This suggests that the numerous defects induced by Au deposition result in a dense distribution of nucleation sites on MoS_2_. As seen in the SEM images (Figure 2E, F) of optoelectronic devices with vdW‐contacted 1T′‐WTe_2_/MoS_2_ and Au/MoS_2_ structures, the vdW contact method reduces the density of surface defects in MoS_2_ films. This is attributed to the weak vdW interactions, which result in low surface activation energy (E_a_), longer surface diffusion lengths, and reduced diffusion barriers.

Raman spectroscopy further confirms the increased surface defect density caused by sputtered Au deposition onto MoS_2_ films [28, 29], as shown in Figure 2G. After deposition, the A_1g_ and E^1^_2g_ mode intensities of the Au/MoS_2_ films are significantly reduced, and the full width at half maximum (FWHM) values increase compared with those of pristine MoS_2_ films, indicating a higher defect density.

Fermi level pinning is a key factor limiting the modulation of Schottky barrier height, making precise control challenging. The effective contact barrier height of deposited Au/MoS_2_ junctions is lower than the theoretical Schottky barrier height predicted by the Schottky–Mott relation. This effective barrier height can be extracted from temperature‐dependent *I*_ds–_ *V*_ds_ curves of deposited Au/MoS_2_/Au field‐effect transistors (FETs) [30, 31], as shown in Figure 2H.

The drain current density (*I*_ds_, in µA µm^−1^), thermally injected from the metal contact into the 2D channel through a reverse‐biased Schottky barrier, can be described by the 2D thermionic emission equation [30, 32]:

(1) $$I_{ds}=AA_{2D}^{*}T^{3/2}\exp\left[-\frac{q}{\kappa_{B}T}\left(\Phi_{SB}-\frac{V_{ds}}{n}\right)\right]$$

where *A* is the Schottky junction contact area, *A* ^*^_2D_ is the 2D equivalent Richardson constant, *q* is the elementary charge, *κ*_B_ is the Boltzmann constant, *ϕ*_SB_ is the effective contact barrier height to be determined, *V*_ds_ is the source–drain voltage, and *n* is the ideality factor.

Simplifying the 2D thermionic emission equation yields a linear Arrhenius plot of ln (*I*_ds_/*T* ^3/2^) vs. 1,000/*T*, where *T* is the temperature (Figure 2I). The effective contact barrier height of deposited Au/MoS_2_ is extracted as ∼413 meV from the slope of this plot (Figure 2J). In contrast, the ideal Schottky barrier height predicted by the Schottky–Mott relation is 790 meV, highlighting the significant role of Fermi level pinning in deposited Au/MoS_2_ junctions.

2D optoelectronic devices were fabricated using vdW contact strategies to address both surface defects and Fermi level pinning. Devices based on high‐barrier (vdW‐contacted Au/MoS_2_) and low‐barrier (1T′‐WTe_2_/MoS_2_) structures exhibit defect‐free surfaces, as previously shown in SEM images. Energy dispersive spectroscopy (EDS) mapping confirms a uniform distribution of the MoS_2_ and 1T′‐WTe_2_ across the device, *i.e*., Mo, S, W, and Te signals are homogeneously present as shown in Figure S2. Atomic force microscopy (AFM) reveals flake thicknesses of 3.20 nm for MoS_2_ and 20.41 nm for 1T′‐WTe_2_ (Figure S3). As shown in Figure S4, the Raman peaks of 1T′‐WTe_2_, namely ^3^A_2_, ^4^A_1_, ^8^A_1_, and ^10^A_1_, appear near 111.7, 131.8, 162.8, and 210.1 cm^−1^, respectively, consistent with its known crystal structure [33]. Raman spectra of MoS_2_ exhibit an E^1^_2g–_A_1g_ peak separation of 24.8 cm^−1^, consistent with the measured thickness [34, 35]. Moreover, after the vdW contact with the bottom of the Au electrode, the positions and linewidths of the Raman characteristic peaks did not change significantly, indicating that the metal contact did not introduce noticeable structural disturbances or defects. These results confirm that vdW‐contacted surfaces are extremely clean, with minimal defects, making them suitable for near‐ideal Schottky interfaces. Such surfaces minimise Fermi level pinning and enable precise barrier height modulation [30, 36].

The vdW‐contacted Au/MoS_2_ and 1T′‐WTe_2_/MoS_2_ structures yield high and low barriers, respectively, both consistent with the Schottky–Mott relation. Calculations based on the 2D thermionic emission equation determine an effective barrier height of 754 meV for vdW‐contacted Au/MoS_2_, as shown in Figure 2K and Figure S5, closely matching the theoretical value. For 1T′‐WTe_2_/MoS_2_, the effective barrier height is 473 meV, as shown in Figure 2L and Figure S6, aligning with the calculated Schottky–Mott value of ∼490 meV. These findings strongly validate the vdW contact strategy as an effective approach to minimise defects and suppress Fermi level pinning.

### 2.3 PPC Enhanced by Higher Barriers in Photomemory

**FIGURE 3:**
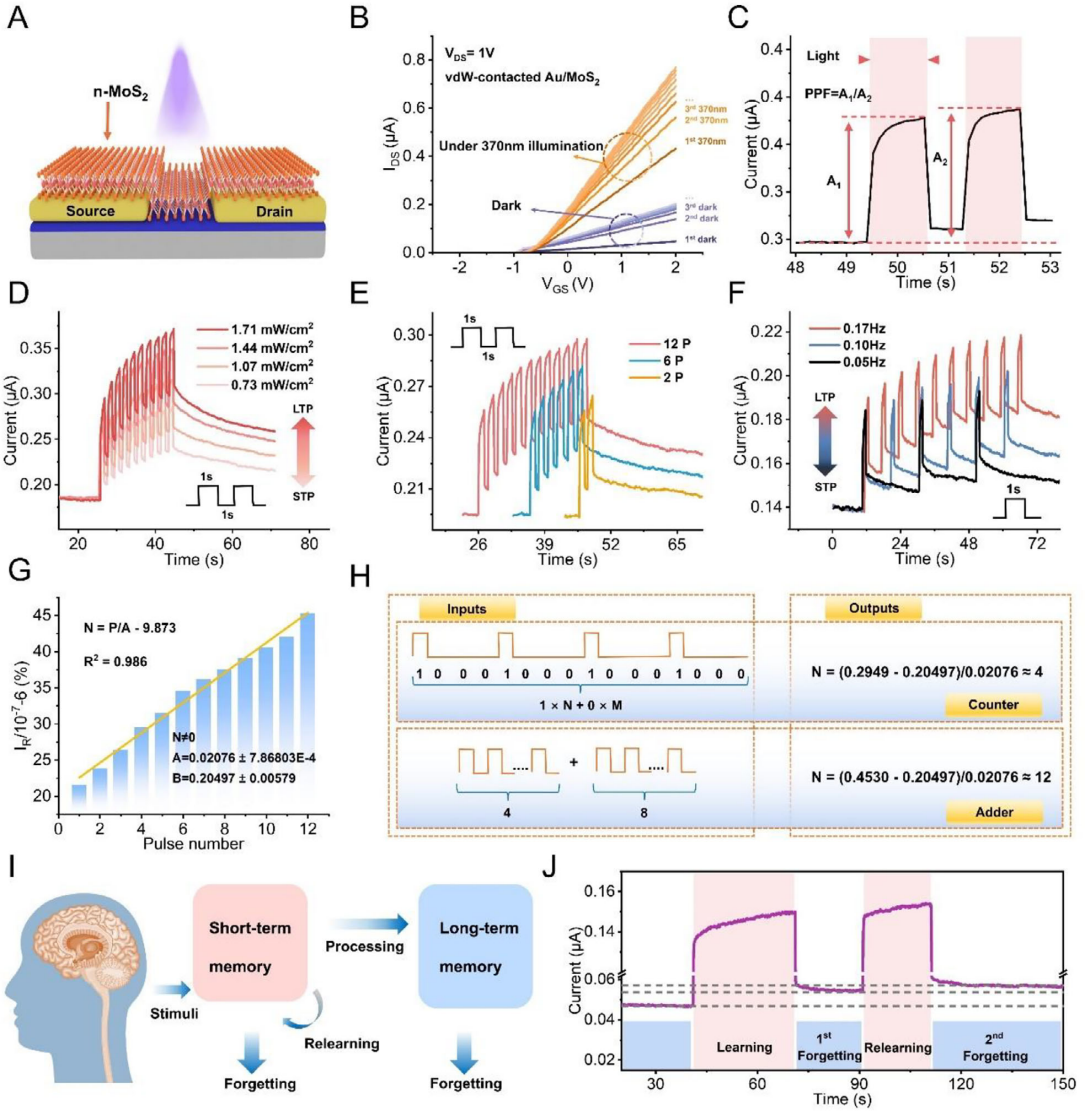
PPC enhanced by Higher Barriers in Photomemory. (A) Side view of the proposed vdW‐contacted Au/MoS_2_ photomemory. (B) Dark–light test of the vdW‐contacted Au/MoS_2_ photomemory. (C) Photocurrent response triggered by a pair of light pulses. Transition from STP to LTP induced by increasing the (D) intensity (pulse width: 1 s; interval: 1 s), (E) number (pulse width: 1 s, interval: 1 s), and (F) frequency (pulse width: 1 s) of pulsed light stimuli. (G) Linear increase in relaxation current change with the number of pulses. (H) Schematic of counting and adding operations performed by the device. (I) Schematic of memory formation in the human brain. (J) Measured synaptic weight recovery, demonstrating that reduced pulse stimulation can restore the channel conductance after spontaneous decay.

The higher Schottky barrier height of the vdW‐contacted Au/MoS_2_ structure paves the way for photomemory applications. Figure 3A shows the schematic of the vdW‐contacted Au/MoS_2_ photomemory, where mechanically exfoliated few‐layer MoS_2_ flakes serve as the channel material bridging the Au electrodes. The presence of a high Schottky barrier height benefits charge retention, which is critical for reliable photomemory operation. As shown in Figure 3B, under identical gate voltage *V*_g_ and bias voltage *V*_ds_, the current increases significantly with the number of cycles, indicating reduced resistance and enhanced conductivity. Moreover, the vdW‐contacted Au/MoS_2_ photomemory exhibits excellent charge retention, with PPCG reaching ∼307.6% after repeated cycles (Figure S7). This enhancement is attributed to the higher Schottky barrier height at the contact interface, which effectively suppresses carrier recombination and extends retention time. Such a high PPCG value highlights the significant potential of the device for advanced photomemory applications.

The higher PPCG also enables simulation of optoelectronic synapses exhibiting plasticity. Biological learning and memory depend on synaptic plasticity, which can be classified into short‐term plasticity (STP) or long‐term plasticity (LTP) based on duration [37, 38]. Paired‐pulse facilitation (PPF), a hallmark of STP in biological synapses [39, 40], is demonstrated in Figure 3C, where the PPF index is defined as *A*_2_/*A*_1_, with *A*_1_ and *A*_2_ being the photocurrents induced by the first and second light pulses, respectively. Figure S8A presents current responses to repeated light pulses at fixed intervals (Δ*t*), where the ratio of the photocurrent generated by the *n* ^th^ pulse (*A*_n_) to that of the first pulse (*A*_1_) is used to quantify contrast. The gradual increase in synaptic weight with successive pulses mimics the dynamic high‐pass filtering observed in biological signal processing (Figure S8B) [41].

The transition from STP to LTP is achieved by adjusting the intensity, number, or frequency of light pulses. As shown in Figure 3D, increasing the light intensity from 0.73 to 1.71 mW/cm^2^ using ten light pulses enables a transition from STP to LTP. Similarly, increasing either the number (Figure 3E) or frequency (Figure 3F) of pulses results in the same transition. Further analysis of multilevel storage (Figure S9A) reveals that the relaxation current increases linearly with the number of pulses, with a linearity (R^2^) of 0.986 (Figure S9B) [42].

Owing to the high linearity of the relaxation currents, the device shows strong potential for computational functions such as arithmetic and logic operations [43, 44]. As shown in Figure 3G, a linear relationship *N* = (*P*‐*B*)/*A* was established, where *N* is the number of light pulses, *P* is the simplified value of the relaxation current, *B* is a constant (0.20497), and *A* is the simplified photocurrent increment per light pulse. When the relaxation current of the photomemory device reaches 629.49 nA, the total number of applied light pulses, four in this case, can be accurately determined (upper panel of Figure 3H). Similarly, when bundles of four and eight light pulses are applied sequentially, the device produces a relaxation current of 645.3 nA, equivalent to the photocurrent generated by 12 light pulses. This demonstrates that a basic addition operation can be realised using the photomemory device (lower panel of Figure 3H).

Additionally, the device mimics the relearning facilitation phenomenon observed in biological memory, where re‐establishing a synaptic weight requires less stimulation than the initial potentiation (Figure 3I). As shown in Figure 3J, a 30 s light pulse gradually increased the photomemory current. Upon removal of the stimulus, spontaneous decay of the current occurred, simulating memory loss. However, when the stimulus was reapplied, the current level previously induced by the first pulse was restored within 20 s, and subsequent decay was suppressed. These simulations of brain‐like behavior highlight the potential of photomemory devices for neuromorphic applications [41, 45, 46, 47].

### 2.4 PPC Inhibited by Lower Barriers in Photodetectors

**FIGURE 4:**
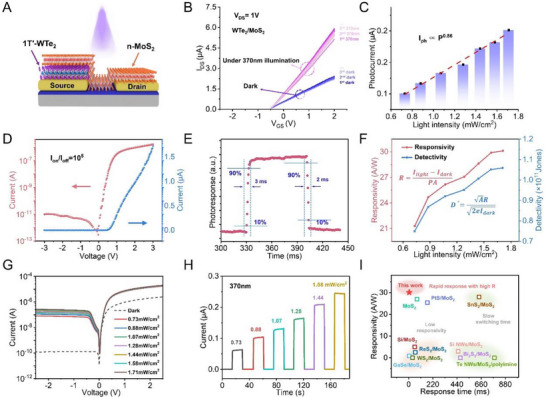
PPC inhibited by lower barriers in photodetectors. (A) Side view of the proposed vdW‐contacted 1T′‐WTe_2_/MoS_2_ heterojunction photodetector. (B) Dark–light test of the 1T′‐WTe_2_/MoS_2_ photodetector. (C) Functional relationship between photocurrent and light intensity (370 nm). (D) *I*_ds_−*V*_ds_ curves of the heterojunction on logarithmic and linear scales. (E) Rise and fall times of the photoresponse. (F) Responsivity and detectivity under different light intensities. (G) *I*_ds_−*V*_ds_ curves under 370 nm illumination at various light power densities. (H) Real‐time photoresponse of the device under varying light intensities. (I) Comparison of response time, responsivity, and detectivity of the 1T′‐WTe_2_/MoS_2_ photodetector with previously reported devices.

A lower Schottky barrier height can effectively suppress PPC and enhance signal reliability in photodetection. Figure 4A presents a schematic of the fabricated 1T′‐WTe_2_/MoS_2_ photodetector, in which the vdW heterojunction comprises semimetallic 1T′‐WTe_2_ and semiconducting MoS_2_, with MoS_2_ acting as the light‐absorbing layer. Compared with the vdW‐contacted Au/MoS_2_ junction, the 1T′‐WTe_2_/MoS_2_ junction forms a lower Schottky barrier height due to the closer match between the work function of 1T′‐WTe_2_ and the electron affinity of MoS_2_. This reduced barrier height modulates carrier transport behavior, suppressing PPC and thereby improving current signal stability in the 1T′‐WTe_2_/MoS_2_ photodetector, as illustrated in Figure 4B. The current values for both device types (vdW‐contacted Au/MoS_2_ and 1T′‐WTe_2_/MoS_2_), extracted from their transfer curves, are shown in Figure S10, offering a direct comparison of their behaviors. Notably, the cycle‐to‐cycle variation in the 1T′‐WTe_2_/MoS_2_ device's dark current is much smaller than that of the Au/MoS_2_ device, indicating significantly greater stability. This enhanced stability leads to improved photocurrent reliability, reflecting the advantages of the 1T′‐WTe_2_/MoS_2_ structure for photodetector applications.

To further investigate the operational stability of the 1T′‐WTe_2_/MoS_2_ photodetector under repeated light excitation, the PPCG was tested over 180 consecutive dark–light cycles under the same environmental conditions. As shown in Figure S11, the PPCG remained consistently low with an average value of 4.72% and showed no significant cumulative drift throughout the measurement. This value is over an order of magnitude lower than that of the high‐barrier Au/MoS_2_ device (which was ∼307.6%). It should be noted that PPCG is a relative metric with respect to the absolute value of initial dark current, so it can reflect the effective memory contrast under the given operating conditions of the device. However, to determine whether this memory behavior originates from barrier engineering rather than external interface effects, we conducted detailed forward and backward gate voltage scans to study the hysteresis behavior of the transfer characteristics in these two types of devices. As shown in Figure S12, under the same scanning conditions, devices with different structures fabricated on the same Si/SiO_2_ substrate exhibited similar hysteresis behavior, indicating that the influence of the channel and gate dielectrics interfaces is common to all devices and is insufficient to explain the differences in PPC behavior.

In addition, we also analyzed the current relaxation dynamics after photoexcitation removal. As shown in Figure S13, the photocurrent of the 1T′‐WTe_2_/MoS_2_ photodetector rapidly returns to the baseline within the millisecond regime, whereas the Au/MoS_2_ device exhibits a significantly extended relaxation process, lasting several minutes. The pronounced difference in relaxation time constants provides direct evidence of fundamentally distinct carrier storage and recombination dynamics in the two devices, fully consistent with the trend revealed by PPCG. A lower PPCG not only indicates enhanced signal stability and rapid recovery to the baseline state but also reflects a stable dark current and reduced light‐induced noise. These features significantly enhance detection accuracy and long‐term reliability, making the device particularly suitable for weak light detection and high‐stability sensing applications.

Benefiting from the low PPCG, the 1T′‐WTe_2_/MoS_2_ photodetector exhibits excellent optoelectronic performance. The response characteristics of this near‐ideal 2D semimetal/semiconductor Schottky junction, under varying light intensities, reveal an almost linear dependence (power‐law exponent ≈ 0.86) between photocurrent and incident light intensity [48] (Figure 4C). Figure 4D presents both linear and logarithmic *I*_ds–_ *V*_ds_ curves in the dark, demonstrating typical rectifying behavior with a maximum rectification ratio of ∼10^5^ at ± 3 V and an ultralow reverse current of 10^−11^ A. These properties enable further investigation into its photoelectric characteristics.

The response speed of a photodetector is critical in determining its ability to track rapidly changing optical signals. The rise time (τ_r_) is defined as the time taken for photocurrent to increase from 10% to 90% of its maximum value, while the fall time (τ_f_) is the time to drop from 90% to 10% [49]. Under periodic 370 nm illumination (Figure 4E), τ_r_ and τ_f_ are measured to be approximately 3 and 2 ms, respectively. These fast dynamics confirm efficient carrier recombination and effective PPC suppression, greatly improving the response speed of the MoS_2_‐based device.

Responsivity (R) and detectivity (D^*^) are also key metrics of photodetector performance, calculated using the following equations:

(2) $$R=\frac{I_{light}-I_{dark}}{PA}$$

(3) $$D^{*}=\frac{\sqrt{A}R}{\sqrt{2eI_{dark}}}$$

where *I*_light_ and *I*_dark_ are the photocurrent and dark current, respectively; *P* is the light power density; *A* is the effective area of the device; and *e* is the elementary charge. As shown in Figure 4F, R increases with light intensity, reaching a maximum of 30.1 A/W at 1.71 mW/cm^2^. D^*^ follows a similar trend, peaking at 1.05 × 10^11^ Jones under the same conditions.

The 1T′‐WTe_2_/MoS_2_ photodetector also demonstrates excellent performance under weak illumination. Figure 4G shows the *I*_ds–_ *V*_ds_ characteristic curve under 370 nm light, with photocurrent increasing steadily as light intensity rises. At *V*_ds_ = −0.5 V, the dark current is 1.27 × 10^−10^ A, and the photocurrent reaches 2.29 × 10^−7^ A under 1.71 mW/cm^2^, confirming strong photoresponse. Current–time (*I–T*) measurements (Figure 4H) further validate this behavior. Even at a relatively low light intensity of 0.88 mW/cm^2^, the device exhibits an on/off ratio exceeding three orders of magnitude.

To benchmark performance, we compared this device with previously reported MoS_2_‐based photodetectors [50, 51, 52, 53, 54, 55, 56, 57, 58, 59]. As shown in Figure 4I, the 1T′‐WTe_2_/MoS_2_ device demonstrates superior R and response speed. These advantages are closely linked to the use of a semimetal to reduce PPC via barrier engineering, making it highly promising for applications requiring fast and sensitive light detection.

### 2.5 Mechanism of Barrier Engineering in Modulating PPC

**FIGURE 5:**
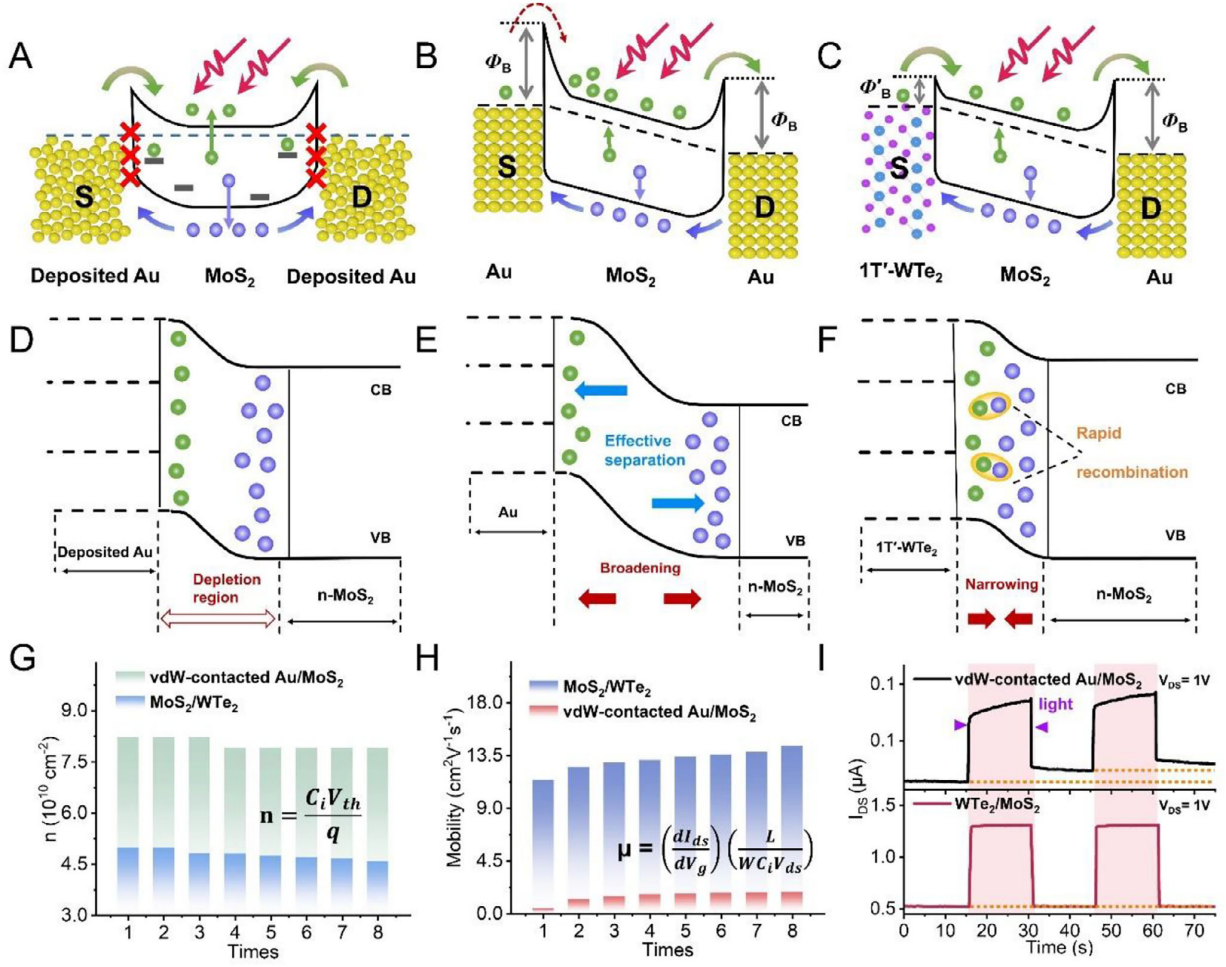
Mechanism of barrier engineering in modulating PPC. Schematic diagrams illustrating the energy band structure and photogenerated carrier transport for (A) deposited Au/MoS_2_, (B) vdW‐contacted Au/MoS_2_, and (C) 1T′‐WTe_2_/MoS_2_. Schematic diagrams showing the depletion region for (D) deposited Au/MoS_2_, (E) vdW‐contacted Au/MoS_2_, and (F) 1T′‐WTe_2_/MoS_2_. Comparison of (G) carrier concentration and (H) mobility in vdW‐contacted Au/MoS_2_ and 1T′‐WTe_2_/MoS_2_ devices under dark conditions. (I), Comparison of dynamic current changes in vdW‐contacted Au/MoS_2_ and 1T′‐WTe_2_/MoS_2_ devices under dark and light conditions.

The mechanism of PPC modulation through barrier engineering was systematically investigated, revealing how the Schottky barrier height influences the transport behavior of photogenerated carriers. Conventionally deposited Au/MoS_2_ contacts lead to Fermi level pinning and the formation of defect states, which disrupt carrier transport and reduce controllability, as illustrated in Figure 5A. In contrast, vdW contact with different electrode materials enables effective barrier engineering. As shown in Figure 5B, vdW‐contacted Au/MoS_2_ devices form higher Schottky barriers on both sides. Even when a positive *V*_ds_ bias is applied, photogenerated carriers must overcome substantial energy barriers to be transported, causing carrier accumulation at the interfaces.

For the 1T′‐WTe_2_/MoS_2_ device, the introduction of a semimetal contact significantly reduces the Schottky barrier height on the 1T′‐WTe_2_/MoS_2_ side. As shown in Figure 5C, under forward bias (*V*_ds_ > 0), the 1T′‐WTe_2_/MoS_2_ junction operates in reverse bias, but *ϕ'*_SB_ is small, allowing easier electron transfer. Simultaneously, the Au/MoS_2_ junction is forward biased, causing the Fermi level of MoS_2_ to rise and the built‐in potential to drop. This further reduces the Schottky barrier height at the MoS_2_ interface, enhancing electron flow from MoS_2_ to Au. It can be seen that the reduced Schottky barrier height and the electric field generated by the bias promote the efficient transport of photogenerated carriers. In addition, 1T′‐WTe_2_ provides highly conductive contact as a semimetal, offering parallel and fast conductive channels for photogenerated carriers [60, 61]. This rapid extraction pathway further shortens the carrier residence time in the MoS_2_ channel. Under the combined effect of these factors, the accumulation of carriers at the interface is effectively alleviated, thereby suppressing PPC.

The depletion region also plays a vital role in carrier transport and recombination. When MoS_2_ is in contact with electrode materials (Au or 1T′‐WTe_2_), differences in Fermi levels drive electron transfer to establish equilibrium. For instance, electron flow from MoS_2_ to Au reduces the carrier concentration at the MoS_2_ interface, forming a depletion region (Figure 5D) [62, 63]. The width of the depletion region is given by

(4) W=2εVbi2εVbieNeN

where *ε* is the dielectric constant of MoS_2_, *N* is the carrier concentration in the MoS_2_ (doping density), *e* is the elementary charge, and *V*_bi_ is the built‐in potential. A larger difference between the work function of the electrode and the electron affinity of MoS_2_ results in a higher Schottky barrier and greater built‐in potential *V*_bi_, increasing the depletion width. As shown in Figure 5E, a wider depletion region generates a stronger internal electric field, promoting rapid electron–hole separation, reducing recombination, and improving collection efficiency. In contrast, a narrower depletion region (Figure 5F) increases the likelihood of carrier recombination, shortening carrier lifetime and enhancing response speed.

The transport and recombination processes of photogenerated carriers are crucial for controlling PPC. The carrier concentration within the semiconductor and the mobility of the device during each dark–light test cycle were calculated using the following equations:

(5) $$n=\frac{C_{i}V_{th}}{q}$$

(6) $$\mu_{eff}=\left(\frac{dI_{ds}}{dV_{g}}\right)\left(\frac{L}{WC_{i}V_{ds}}\right)$$

where *q* is the elementary charge (1.6 × 10^−19^ C), *C*_i_ is the gate capacitance (1.33 × 10^−8^ F cm^−2^ for 285 nm SiO_2_), *V*_th_ is the threshold voltage, and *V*_g_ is the applied back‐gate voltage. As shown in Figure 5G, H, the carrier concentration of the higher‐barrier vdW‐contacted Au/MoS_2_ device remains consistently greater than that of the lower‐barrier 1T′‐WTe_2_/MoS_2_ device under dark conditions, whereas the 1T′‐WTe_2_/MoS_2_ device exhibits higher mobility. Notably, in the vdW‐contacted Au/MoS_2_ device, the higher Schottky barrier causes accumulation and slow recombination of photogenerated carriers within the semiconductor, resulting in a gradual rise and decay of the current before and after illumination. This behavior is accompanied by a pronounced PPC effect, as illustrated in Figure 5I. In contrast, the lower‐barrier 1T′‐WTe_2_/MoS_2_ device exhibits rapid current rise and decay, reaching steady values before and after illumination. This indicates fast carrier transport and recombination, shortened response time, and effective suppression of PPC.

These results confirm that barrier engineering can effectively regulate carrier transport and recombination, thereby modulating PPC. On the one hand, the Schottky barrier height at the device interface governs carrier accumulation: higher barriers promote accumulation, while lower barriers facilitate transport. On the other hand, changes in Schottky barrier height also affect the width of the depletion region, thereby influencing the recombination time of photogenerated carriers and enabling precise control over PPC behavior.

## 3 Discussion

In summary, we have demonstrated that engineering Schottky barrier heights via vdW contacts can effectively regulate carrier transport, thereby modulating PPC and offering new strategies for optimising the performance and functionality of optoelectronic devices. The higher‐barrier vdW‐contacted Au/MoS_2_ photomemory exhibits a PPCG of 307.6% and successfully emulates fundamental optoelectronic synaptic behaviors, including STP/LTP and the ‘learning experience’. Owing to the high linearity (0.986) of its relaxation current, this device also supports arithmetic functions such as counting and addition, advancing the development of highly linear artificial synapses for neuromorphic computing. In contrast, the 1T′‐WTe_2_/MoS_2_ photodetector, incorporating a semimetal to lower the Schottky barrier height, reduces the recombination time of photogenerated carriers in the depletion region, thereby improving response speed. The PPCG of the low‐barrier 1T′‐WTe_2_/MoS_2_ photodetector is suppressed to 4.72%, while maintaining a maximum on/off ratio of 10^5^, a response time of 2 ms, and a R of 30.1 A/W.

## 4 Experimental Section

### 4.1 Device Fabrication

The vdW‐contacted Au/MoS_2_ and 1T′‐WTe_2_/MoS_2_ devices were fabricated using a dry‐transfer method involving polydimethylsiloxane (PDMS) films and a transfer platform. The process was as follows: MoS_2_ and 1T′‐WTe_2_ flakes were first exfoliated from their bulk crystals using blue adhesive film. To facilitate flake selection, the exfoliated layers were transferred onto PDMS via a pressing process. Suitable flakes were identified under an optical microscope. These were then transferred onto SiO_2_/Si substrates, pre‐patterned with Cr/Au (30/100 nm) electrodes, using a micromanipulator‐assisted transfer platform. This method enabled the assembly of vdW‐contacted Au/MoS_2_ and 1T′‐WTe_2_/MoS_2_ heterostructures with optimal flake thickness, morphology, and alignment.

### 4.2 Device Characterisation

Raman spectroscopy was conducted using a Horiba HR Evolution 800 system with a 532 nm illumination at 0.5 mW. Surface morphology and elemental composition were examined using scanning electron microscopy (SEM, Zeiss Gemini Ultra‐55) and energy dispersive spectroscopy (EDS, Bruker XFlash 630). Flake thicknesses were characterised using atomic force microscopy (AFM) with scanning probe microscopy modules.

### 4.3 Optoelectronic Measurements

All electrical and photoelectronic measurements were carried out using a semiconductor parameter analyser (Keithley 4200) in conjunction with a cryogenic probe station (LakeShore TTPX), enclosed within a shielding case under ambient conditions. A 370 nm light‐emitting diode (LED) with adjustable output power (0.01–0.18 mW) served as the excitation source. To enable light‐on/off modulation, the LED was synchronised with an optical chopper. For photoresponsivity calculations, the effective light power was normalised using the following expression: *P* = *P*_0_·device area/spot size or beam area, where *P*_0_ is the LED output power. The photoresponse (current vs. time) was recorded under pulsed illumination.

### 4.4 Extraction of Schottky Barrier Height

A 2D Schottky FET can be modelled as two Schottky diodes connected back‐to‐back, with the drain–source voltage (*V*_ds_) predominantly dropping the reverse‐biased contact. Thus, in an n‐type device, carrier injection was governed by the source contact. The drain current density (*I*_ds_, in µA µm^−1^) injected thermionically over the Schottky barrier was given by the 2D thermionic emission Equation (1). Under the condition *V*_ds_ ≫ *κ*_B_ *T*, Equation (1) simplifies to

$$I_{ds}\approx AA_{2D}^{*}T^{3/2}\exp\left(-\frac{q\phi_{SB}}{\kappa_{B}T}\right)$$

This simplified form enables the extraction of *ϕ*_SB_ by finding the slope of the Arrhenius plot, using the following equation:

lnIdsIdsT3/2T3/2=−qϕSBκBT

Subsequently, *ϕ*_SB_ is extracted using the relation $S_{0}=-\frac{q\phi_{SB}}{1000\kappa_{B}}$ at *V*_ds_ = 0.

## Author Contributions

P.H. and X.Z. contributed equally to this work. P.H., X.Z., B.M., I.J., W.Y., and C.Y. conceived the idea. P.H. and X.Z. carried out the experiments (device fabrication, simulation, and performance characterization) with the help of X.C., Y.W., and D.Z. performed the data analysis. B.M., I.J., W.Y., and C.Y. supervised the project. All authors reviewed the manuscript.

## Funding

This work was supported by the National Natural Science Foundation of China (11974222), National Research Foundation of Korea (RS‐2025‐25440502), and the Shandong Provincial Natural Science Foundation (ZR2023MA087).

## Conflicts of Interest

The authors declare no conflicts of interest.

## Supporting information




**Supporting File**: advs74680‐sup‐0001‐SuppMat.doc.

## Data Availability

The data that support the findings of this study are available from the corresponding author upon reasonable request.
