# Supplementary material for: Modulating Persistent Photoconductivity through Barrier Engineering for High‐performance and Multifunctional Two‐dimensional Optoelectronic Devices
Source: Adv Sci (Weinh). 2026 Mar 3;13(27):e24279. doi: 10.1002/advs.202524279 (PMC13170241; doi:10.1002/advs.202524279)
Supplement: Supplementary file 1 — Supporting File: advs74680‐sup‐0001‐SuppMat.doc. [file ADVS-13-e24279-s001.doc]

**Supporting Information**

**Modulating Persistent Photoconductivity Through Barrier Engineering for High-performance and Multifunctional Two-dimensional Optoelectronic Devices**

Panpan Huo^a1^, Xinhao Zhang^a1^, Xiangyong Cui^a^, Yeming Wang^a^, Dong Zhou^a^, Baoyuan Man^a^, Il Jeon^b,c*^, Won Jong Yoo^b*^, and Cheng Yang^a,b,c,d*^

^a^School of Physics and Optoelectronics, Shandong Normal University, Jinan, 250358, People’s Republic of China;

^b^Department of Nano Engineering, Department of Nano Science and Technology, SKKU Advanced Institute of Nanotechnology (SAINT), Sungkyunkwan University (SKKU), Suwon 16419, Republic of Korea;

^c^SKKU Global Research Center (SGRC), Sungkyunkwan University (SKKU), Suwon 16419, Republic of Korea;

^d^Shandong Provincial Key Laboratory of Light Field Physics and Applications, Jinan, 250014, People’s Republic of China

*Corresponding authors.

E-mail: chengyang@sdnu.edu.cn (Cheng Yang); [yoowj@skku.edu](mailto:yoowj@skku.edu) (Won Jong Yoo); il.jeon@spc.oxon.org (Il Jeon).

^1^ These authors contributed equally to this work.


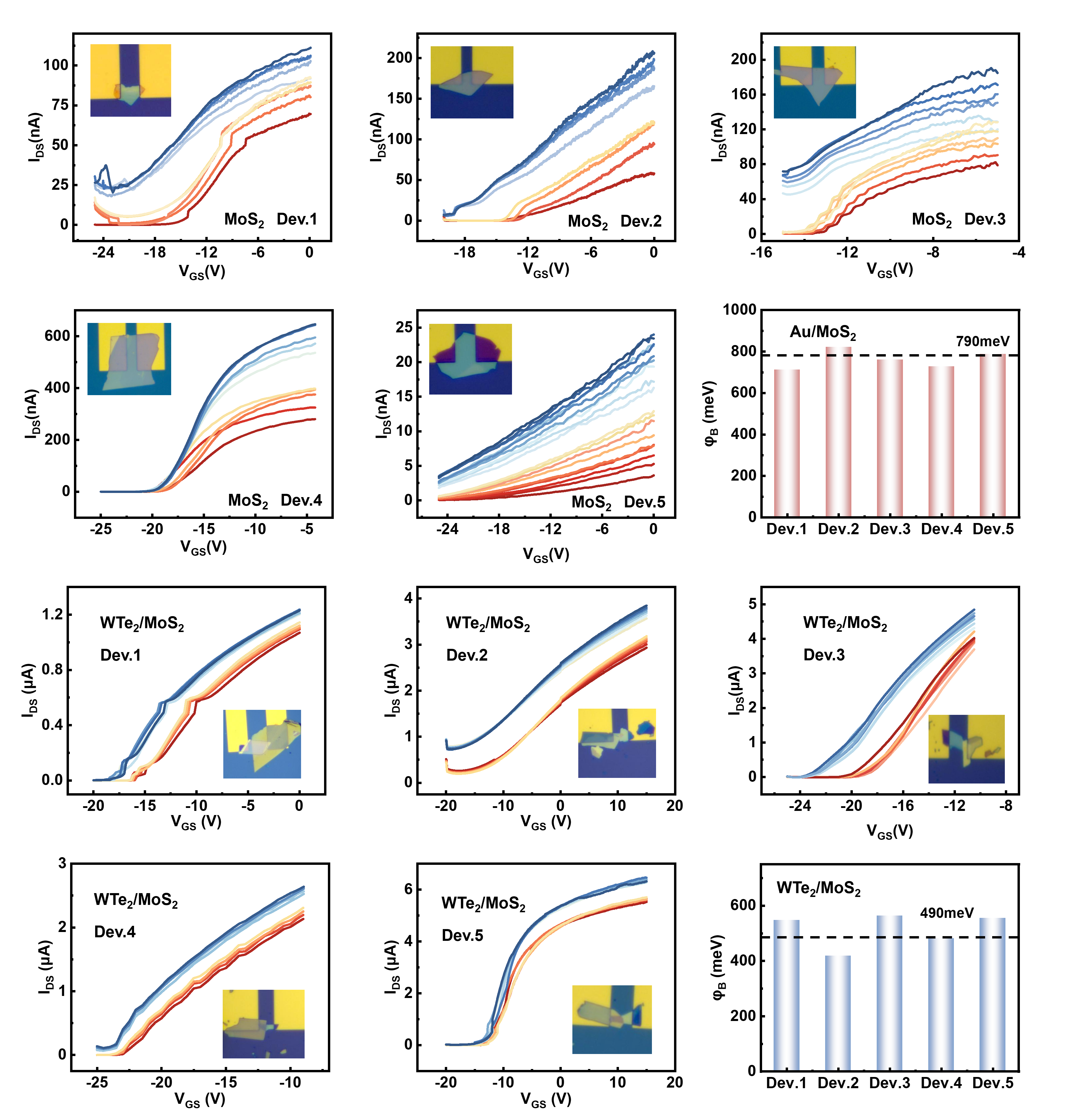


**Figure S1.** Device-to-device repeatability of transfer characteristics and extracted Schottky barrier heights for vdW-contacted Au/MoS_2_ and 1T′-WTe_2_/MoS_2_ devices.

**Figure S2.** A) EDS spectrum of 1T′-WTe_2_/MoS_2_ heterojunction device. B) EDS spectrum of vdW-contacted Au/MoS_2_ device.


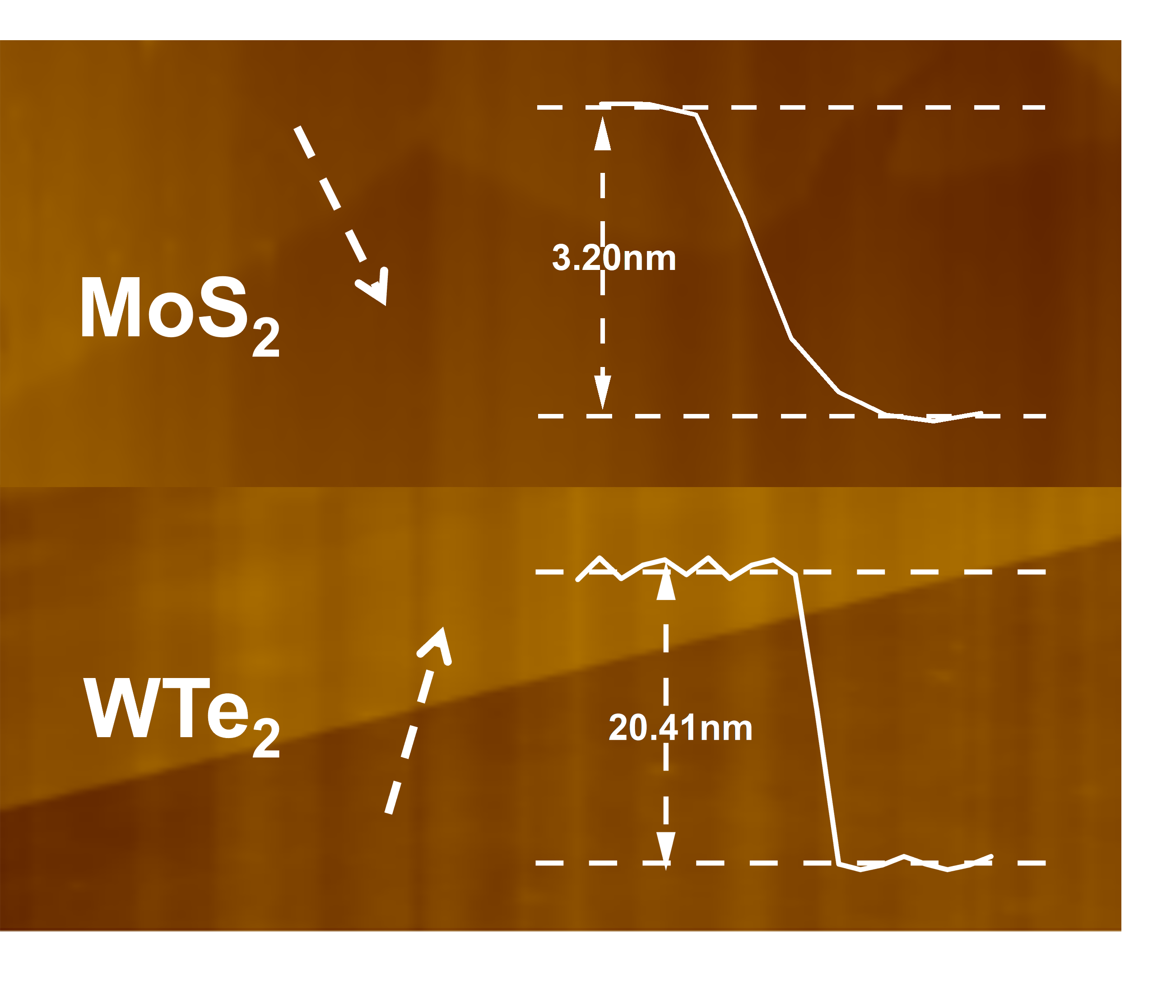


**Figure S3**. AFM images of 1T′-WTe_2_ and MoS_2_.


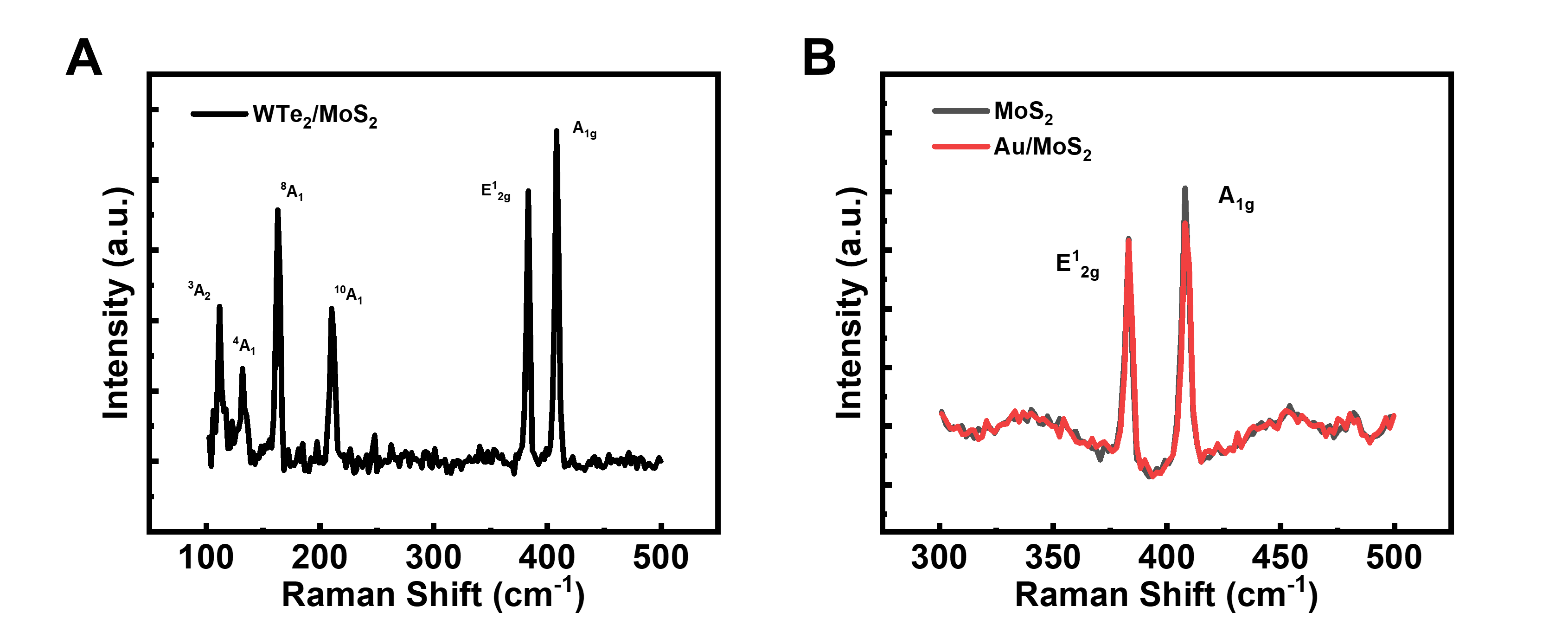


**Figure S4**. Raman spectra of 1T′-WTe_2_/MoS_2_ and Au/MoS_2_.

**
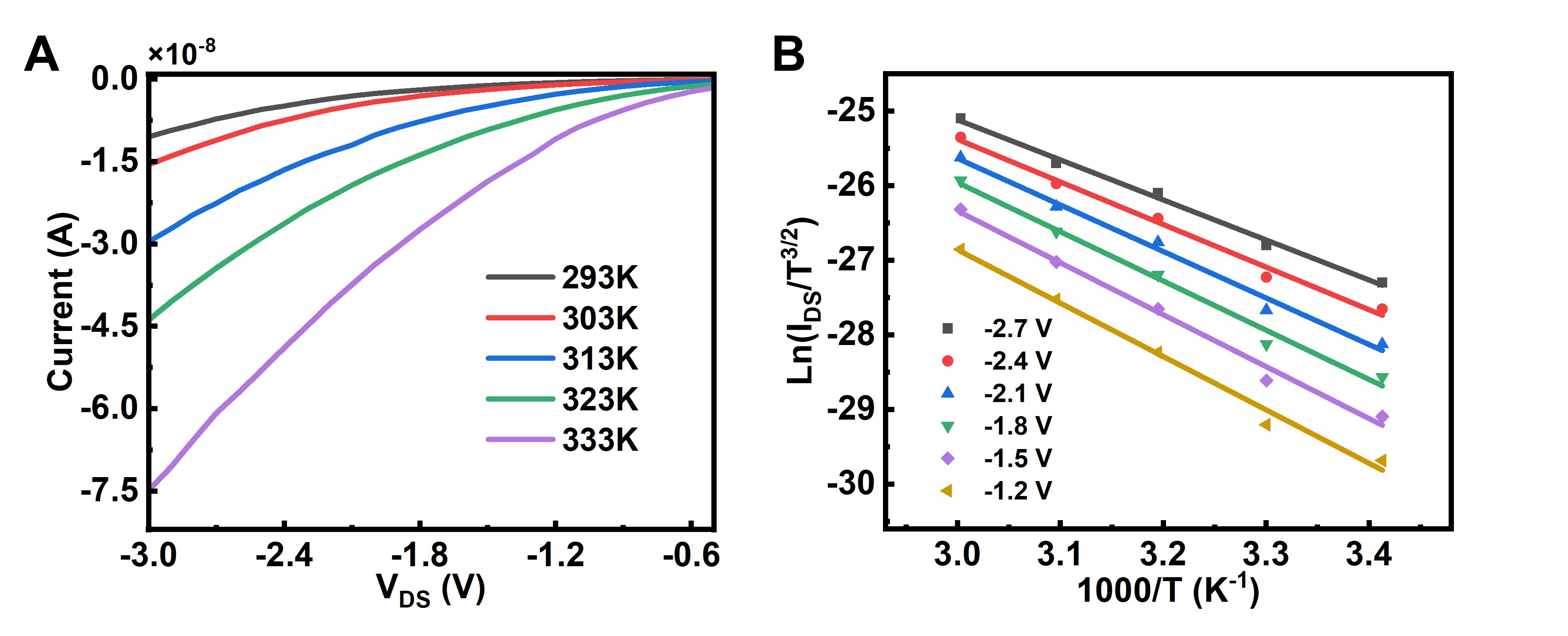
**

**Figure S5.** A) *I*_ds_-*V*_ds_ curves of the Schottky structure vdW-contacted Au/MoS_2_ at different temperatures. B) Linear fitting of the Arrhenius plot for the vdW-contacted Au/MoS_2_ junction at various *V*_ds_.

**
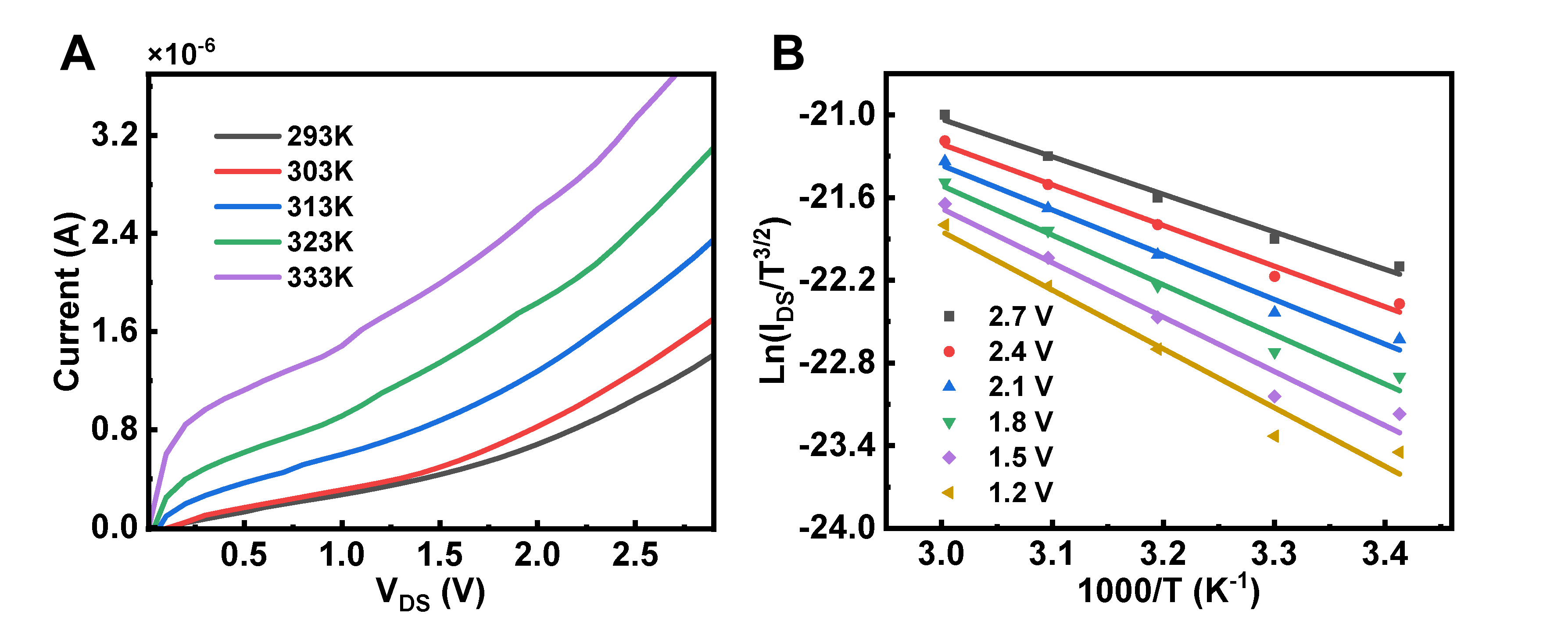
**

**Figure S6.** A) *I*_ds_-*V_d_*_s_ curves of the Schottky structure 1T′-WTe_2_/MoS_2_ at different temperatures. B) Linear fitting of the Arrhenius plot for the 1T′-WTe_2_/MoS_2_ junction at various *V*_ds_.


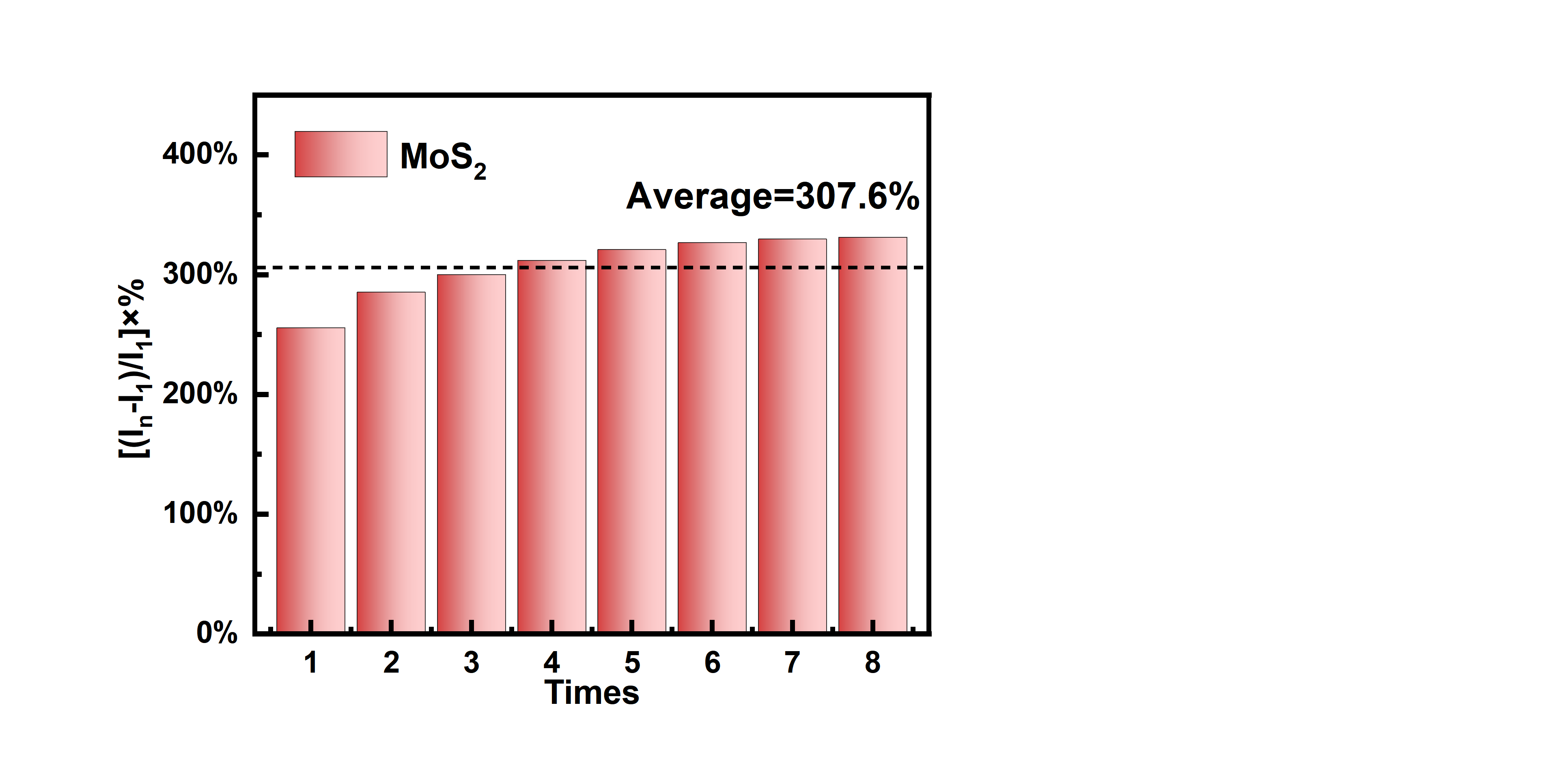
**Figure S7**. PPCG for vdW-contacted Au/MoS_2_ devices

**
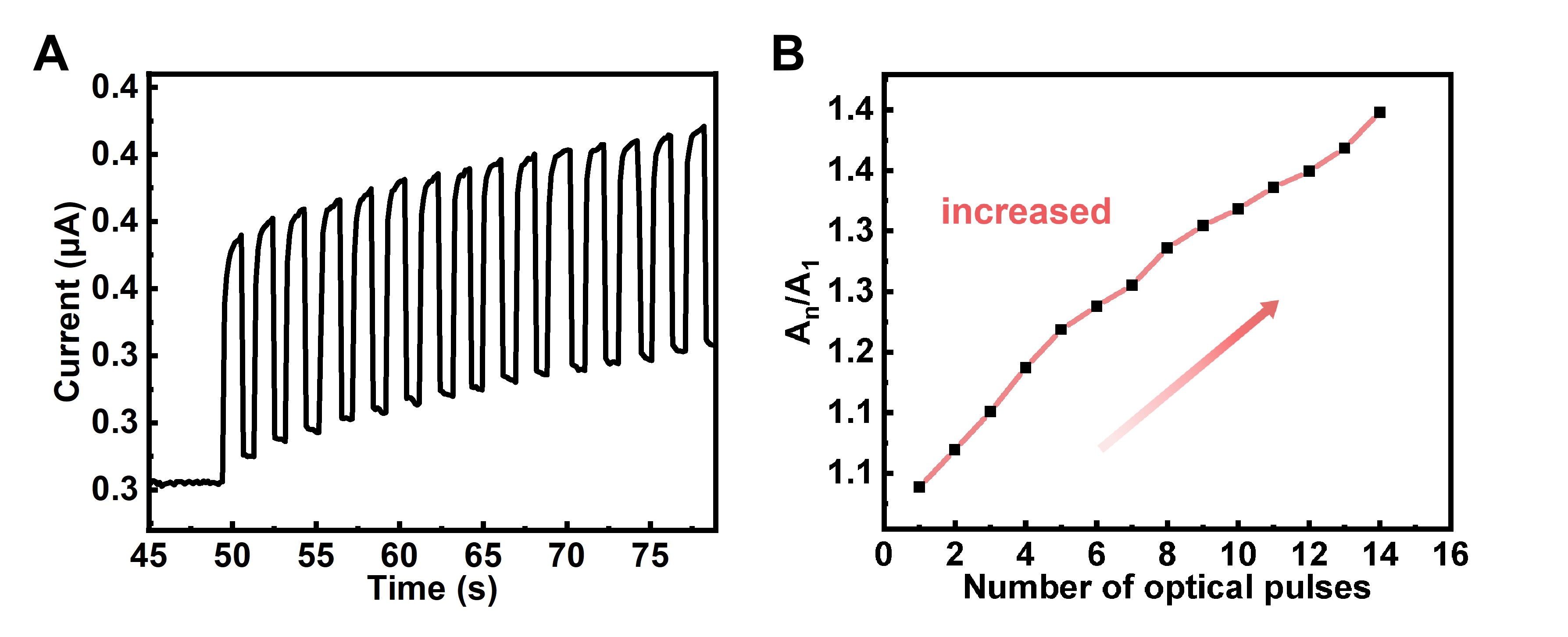
**

**Figure S8.** A) Photocurrent evolution process of multiple pulsed light stimuli. B) The functional relationship between *A*_n_/*A*_1_ and the number of applied light pulses.

**
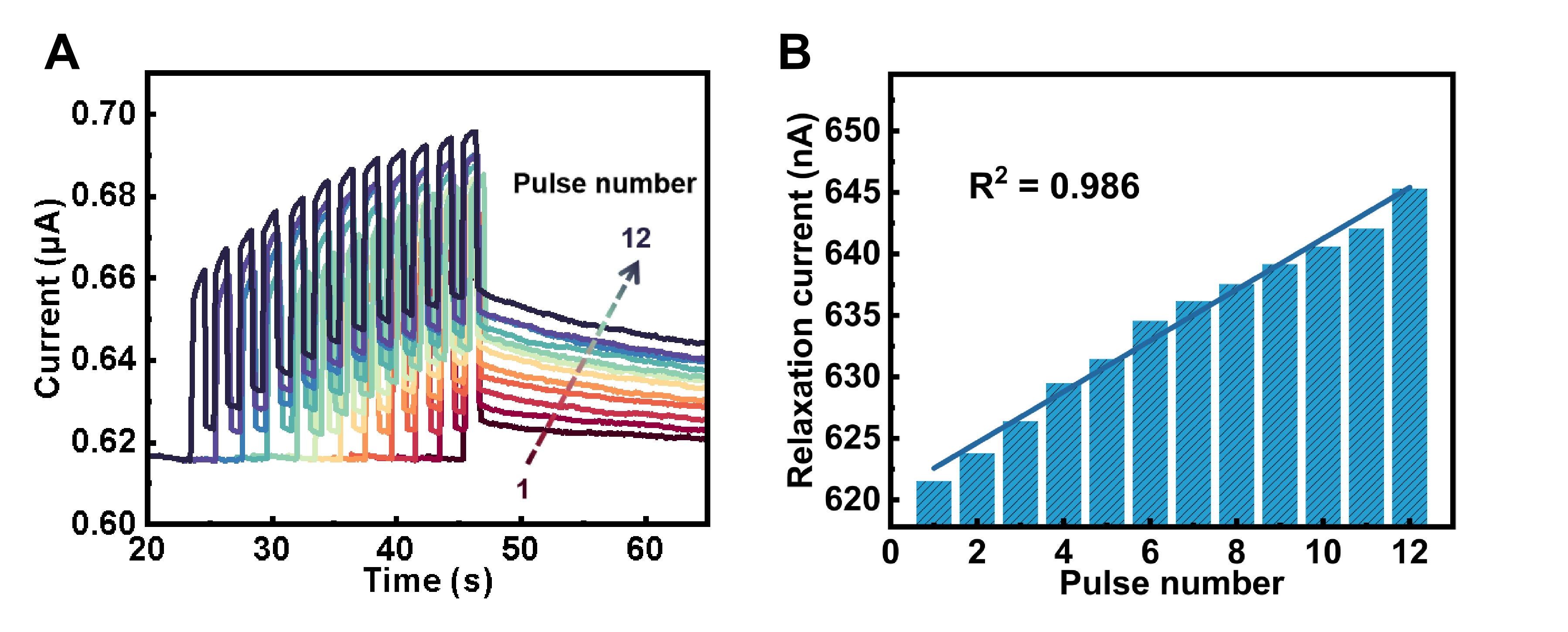
**

**Figure S9.** A) Dependence of the relaxation current of an photo memory device on the number of applied light pulses. B) Fitting analysis of the relationship between relaxation current and pulse number.

**
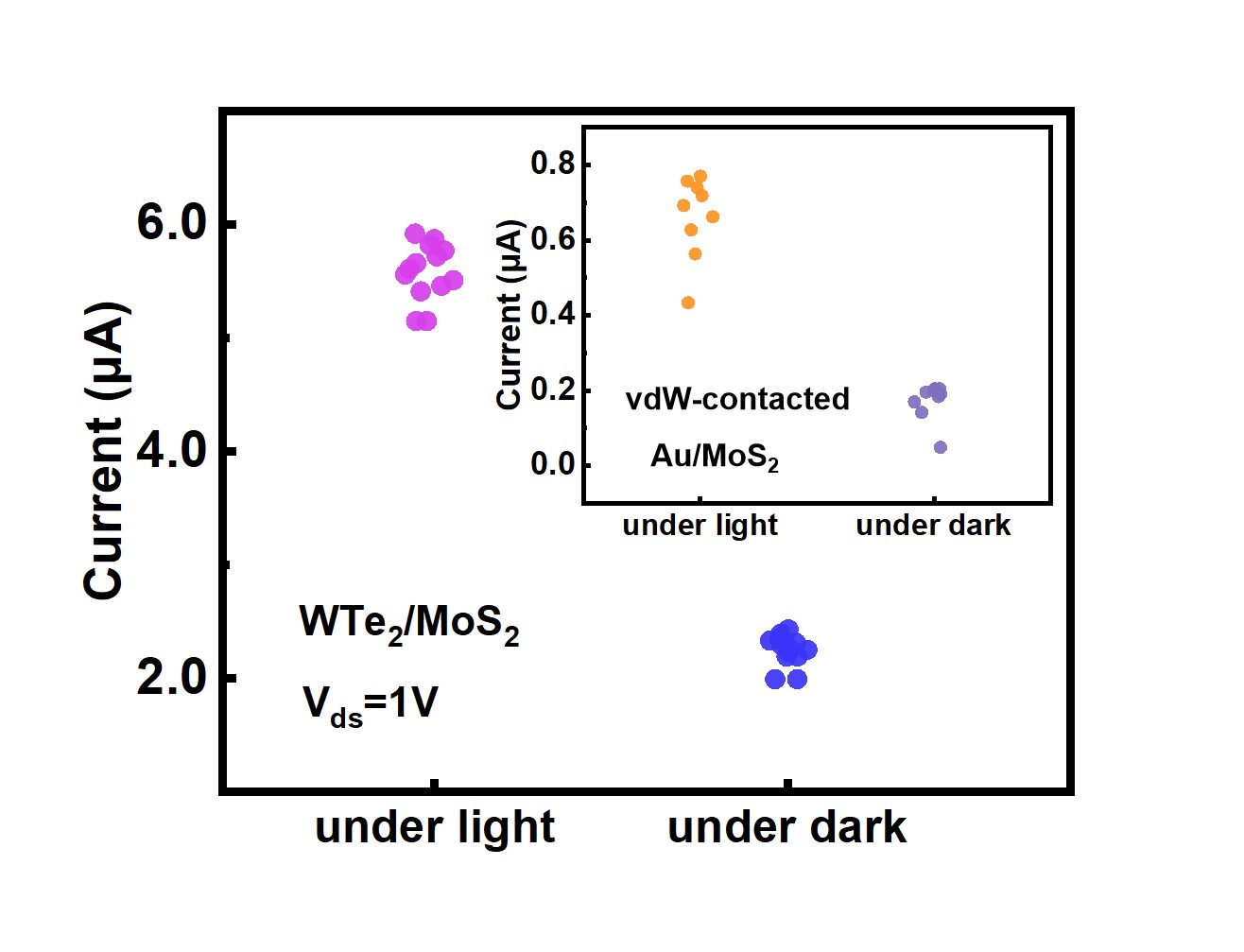
**

**Figure S10**. The current variation of the 1T′-WTe_2_/MoS_2_ detector under light and dark conditions extracted from Fig. 4B. The inset shows the current variation of the vdW-contacted Au/MoS_2_ detector under light and dark conditions.

**
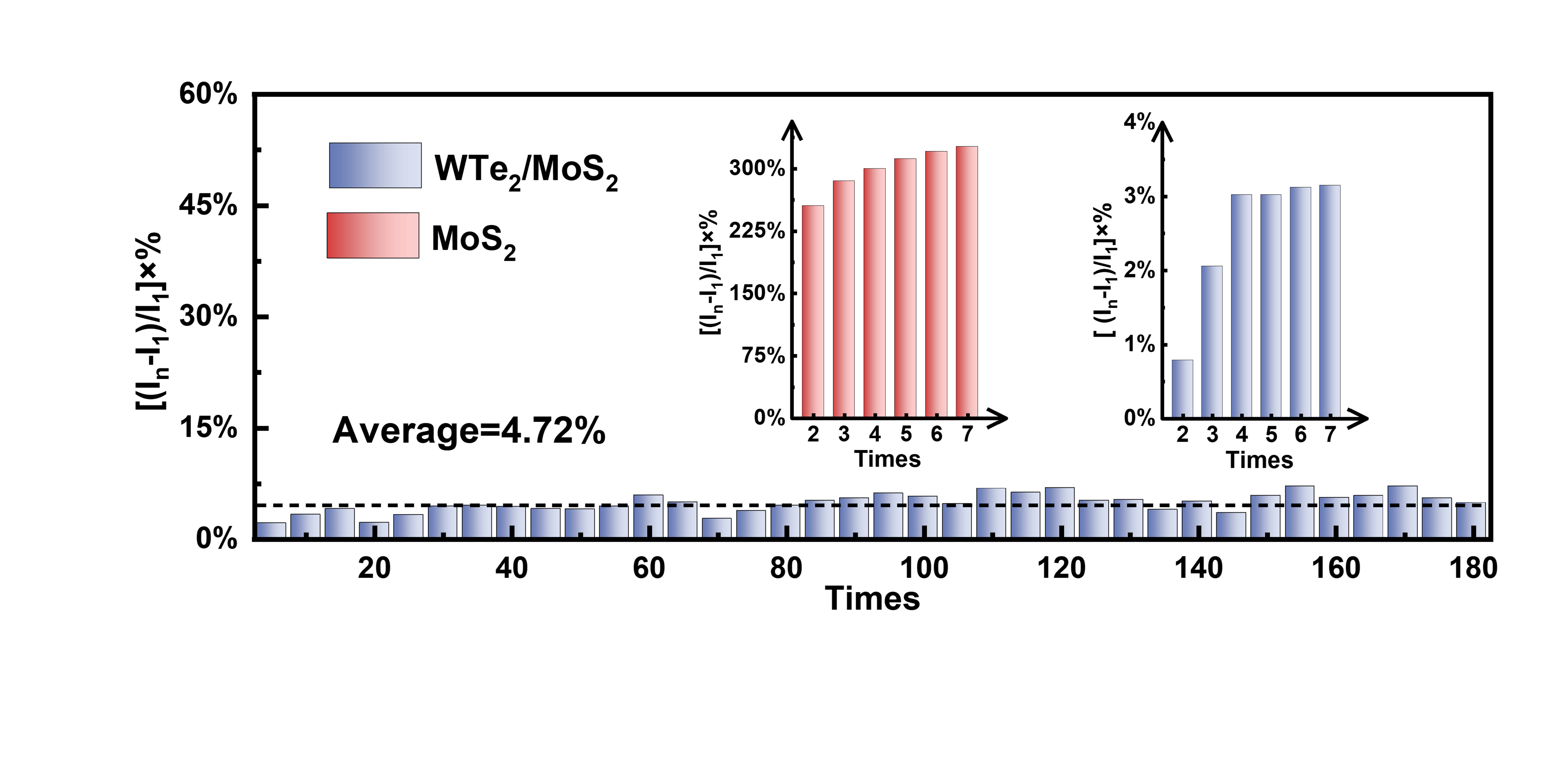
**

**Figure S11**. The long-term stability of 1T′-WTe_2_/MoS_2_ photodetectors in suppressing PPC across multiple cycles. The stability of the vdw-contact 1T′-WTe₂/MoS_2_ device was evaluated through 180 consecutive light-dark cycles under the same environmental conditions. For each cycle, the PPCG was calculated based on the relative dark current compared to the first cycle. The average PPCG was 4.72%, with no significant cumulative drift observed throughout the measurements, indicating stable dark current and PPC behavior under repeated cycling. The inset shows a pronounced difference in the PPC under dark conditions for two structures: vdW-contacted Au/MoS_2_, and 1T′-WTe_2_/MoS_2_.


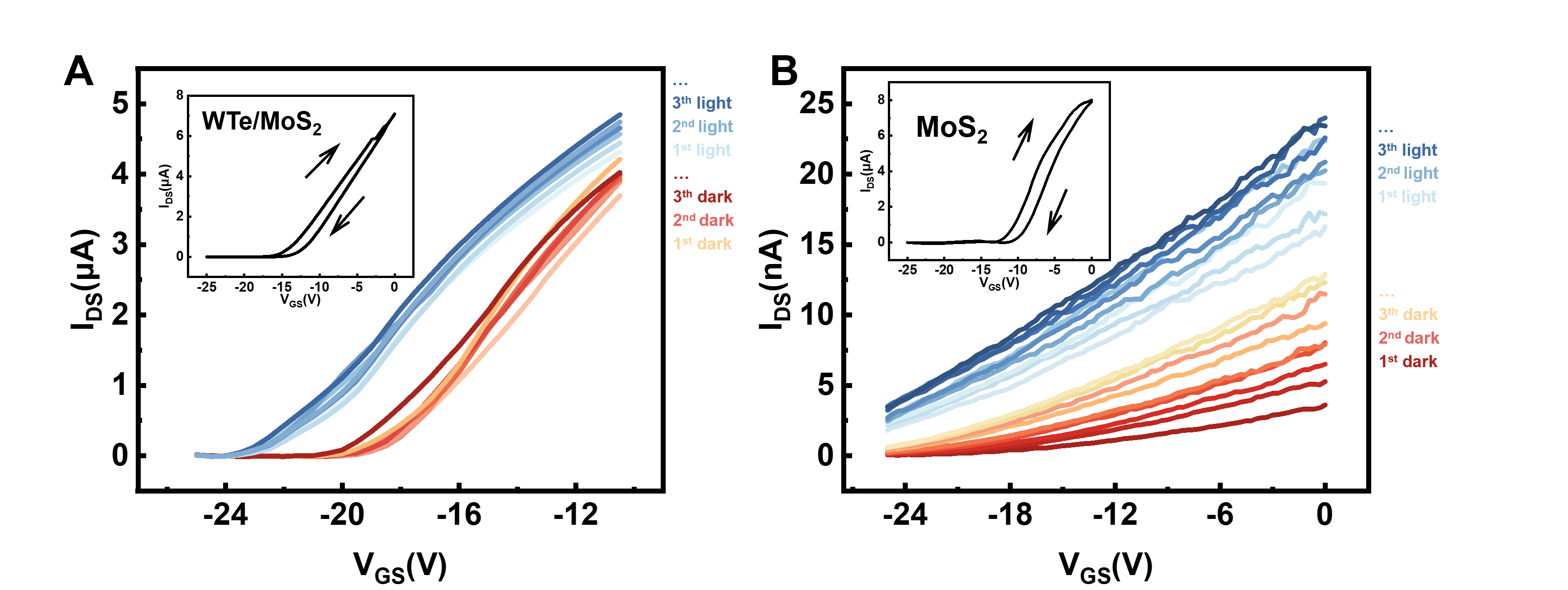


**Figure S12**. Comparison of hysteresis and PPC behavior between vdW-contacted Au/MoS_2_ and 1T′-WTe_2_/MoS_2_ devices.


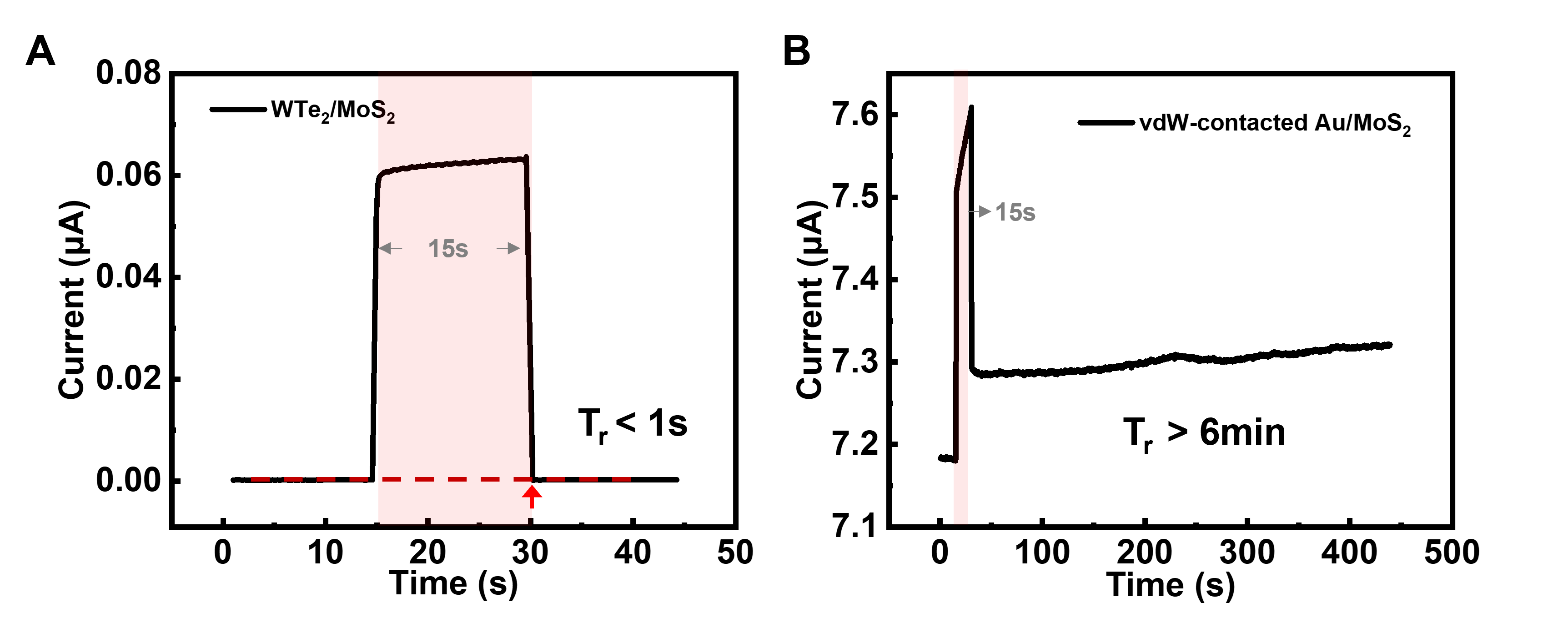
**Figure S13**. Comparison of current relaxation times between vdW-contacted Au/MoS_2_ and 1T′-WTe_2_/MoS_2_ devices.
